# Development and validation of a nomogram for predicting bleeding risk in patients with pulmonary embolism

**DOI:** 10.3389/fmed.2025.1692156

**Published:** 2025-10-09

**Authors:** Tian Ye, Wanlin Lei, Maofeng Wang, Lili Xu

**Affiliations:** ^1^Department of Emergency, Affiliated Dongyang Hospital, Wenzhou Medical University, Dongyang, Zhejiang, China; ^2^Department of Biomedical Sciences Laboratory, Affiliated Dongyang Hospital, Wenzhou Medical University, Dongyang, Zhejiang, China; ^3^Department of Obstetrics, Affiliated Dongyang Hospital, Wenzhou Medical University, Dongyang, Zhejiang, China

**Keywords:** pulmonary embolism, bleeding, risk prediction model, nomogram, adverse effects, anticoagulants

## Abstract

**Purpose:**

Bleeding during anticoagulation therapy represents a critical challenge in pulmonary embolism (PE) management, this study aimed to develop and validate a PE-specific bleeding risk prediction model.

**Methods:**

This retrospective cohort study utilized a clinical research big data platform, including 5,632 hospitalized PE patients (January 2013–December 2024). Significant bleeding within 6 months served as the primary outcome. After excluding variables with >20% missingness, 29 predictors were analyzed. The cohort was randomly split into development (*n* = 3,942) and validation sets (*n* = 1,690). LASSO regression identified key predictors, with multivariable logistic regression constructing the final model. Performance was assessed via AUC-ROC, calibration plots, and decision curve analysis (DCA).

**Results:**

The final model identified six predictors: prior bleeding history, renal insufficiency, red blood cell count, systolic pressure, cerebral infarction, and creatinine. The model demonstrated robust discrimination (development AUC: 0.756, 95%CI: 0.729–0.784; validation AUC: 0.729, 95%CI: 0.685–0.773) and calibration (validation slope: 0.810). DCA confirmed significant net benefit at 5–35% thresholds, with 30% as the optimal cut-off. At this threshold, the model reduced major bleeding by 42% versus standard care.

**Conclusion:**

This novel PE-specific bleeding risk tool provides clinically actionable stratification, enabling personalized anticoagulation intensity adjustment. Implementation may reduce hemorrhage-related morbidity while optimizing resource utilization.

## 1 Introduction

Pulmonary embolism (PE), the third leading cause of cardiovascular mortality after stroke and myocardial infarction (1), remains a critical medical challenge. Recent advances in management—including catheter-directed thrombolysis, mechanical thrombectomy, extracorporeal membrane oxygenation (ECMO), and surgical embolectomy—have expanded therapeutic options (2). Nonetheless, hemorrhage persists as a major complication, especially following thrombolytic therapy (3). Although anticoagulation and thrombolysis are effective in reducing thrombotic burden, they concomitantly increase bleeding risk (4). Accurate prediction of hemorrhagic events is therefore essential for balancing thromboembolic protection against bleeding hazards and guiding personalized treatment strategies.

Accurate prediction of bleeding risk is essential in anticoagulated patients with PE, prompting the development of several predictive models and scores (5, 6). Among these, the PE-SARD score was specifically designed for acute PE and demonstrated a C-index of 0.654 for 30-day major bleeding in a large external validation cohort, outperforming both BACS and PE-CH models (7). The VTE-BLEED score, widely validated in venous thromboembolism, effectively identifies patients at high risk of major bleeding—including intracranial and fatal events—during anticoagulation (8), and retains predictive power over the long term (9). The IMPROVE bleeding score has proven valuable in predicting hemorrhage in high-risk populations such as patients with advanced gastrointestinal cancer (10) and hospitalized COVID-19 patients (11). Machine learning approaches have also shown promise; one model for cancer-associated thrombosis outperformed conventional CAT-BLEED scores (12), and another incorporating liver function markers with PE-SARD improved early bleeding prediction in acute PE (13).

Despite these efforts, commonly used clinical scores such as HAS-BLED and ATRIA were not originally developed or adequately validated in PE populations, leading to limited predictive accuracy in this group. There remains a pressing need to develop or validate dedicated prediction tools tailored specifically to patients with PE.

## 2 Methods

### 2.1 Study population

This retrospective study utilized data from a clinical research big data platform of Affiliated Dongyang Hospital of Wenzhou Medical University. Inclusion criteria for participants were: (1) age over 18 years; (2) discharge diagnosis of pulmonary embolism. Exclusion criteria: (1) Pregnant or lactating women; (2) Patients with incomplete medical histories or examination test results; (3) Patients with missing data of PE or lacking relevant bleeding records; (4) Individuals who died during hospitalization. We identified and included 5,632 patients hospitalized with a confirmed diagnosis of PE between January 2013 and December 2024. Based on bleeding outcome, patients were categorized into two groups: those who experienced significant bleeding (bleeding group, *N* = 447) and those who did not (no bleeding group, *N* = 5,185). The study initially collected data on 32 candidate predictor variables (indicators) potentially associated with bleeding risk. Three variables (weight, weight, BMI) were excluded prior to model development due to a high proportion (>20%) of missing values. Thus, the analysis proceeded with 29 variables. The final cohort of 5,632 patients was randomly partitioned into a training set (*N* = 3,942, 70%) for model training and a testing set (*N* = 1,690, 30%) for subsequent internal validation of the derived risk prediction models. The study protocol received ethics approval from the Ethics Committee of Affiliated Dongyang Hospital of Wenzhou Medical University (approval #2025-YX-157). Informed consent was waived for this study. Prior to conducting the analysis, all patient medical information was anonymized and de-identified.

### 2.2 Outcome definition

The primary outcome of this study was the occurrence of any documented clinically significant bleeding event within 6 months following the diagnosis of PE. In our study, bleeding events were identified based on the presence of any hemorrhagic diagnosis within the primary discharge diagnoses. Bleeding events included gastrointestinal bleeding, intracranial hemorrhage, urinary bleeding, oral bleeding, ophthalmic hemorrhage, and other major bleeds (14). For analysis, outcomes were defined as binary: presence of any qualifying bleeding event (positive outcome) versus absence of bleeding (negative outcome).

### 2.3 Candidate predictor variables

The variables extracted from our hospital’s EMRs were meticulously selected based on their established relevance in existing bleeding risk scores, supporting evidence from the literature, and clinical experience pertinent to bleeding risk in PE patients. (1) Demographics and vitals: Age, height, weight, BMI, systolic blood pressure, diastolic blood pressure. (2) Comorbidities and history: Smoking status, alcohol consumption, diabetes, hypertension, pulmonary hypertension, pulmonary infarction, history of prior bleeding, arterial thrombosis, active malignancy, myocardial infarction, cerebral infarction, renal insufficiency. (3) Treatments: Anticoagulant use, thrombolytic therapy, antiplatelet therapy. (4) Laboratory parameters (measured within 1 month prior to PE diagnosis): white blood cell count (WBC), creatinine, activated partial thromboplastin time (APTT), international normalized ratio (INR), prothrombin time (PT): highest recorded value. Platelet count (PLT), red blood cell count (RBC), hemoglobin (HGB): lowest recorded value. All comorbidities and historical conditions were recorded only if documented before the diagnosis of PE.

### 2.4 Data pre-processing

Data extracted from the clinical research big data platform underwent rigorous preprocessing. Variables with >20% missing values (e.g., height, weight, BMI) were excluded from analysis. For remaining missing values in candidate predictors, multiple imputation by chained equations (MICE) was employed (15, 16). We performed 20 iterations using predictive mean matching as the imputation model, with a random seed set for reproducibility. As part of the data cleaning process, outliers were identified and removed in accordance with conventional criteria for biological plausibility and statistical extremes (values beyond Q3 + 1.5 × IQR or below Q1 − 1.5 × IQR). The cohort was then randomly split in a 7:3 ratio stratified into a training set (70%) for model training and a validation set (30%) for performance evaluation.

### 2.5 Model building

Feature selection was performed using least absolute shrinkage and selection operator (LASSO) regression (17) with 10-fold cross-validation to identify optimal predictors while mitigating overfitting. The lambda.1se value was chosen to select the final model. Variables retained at the optimal lambda value were subsequently entered into multivariable logistic regression. Significant indicators identified in the univariate analysis were assessed for multicollinearity using variance inflation factors (VIFs), with a threshold of VIF <10 indicating no severe multicollinearity. The linearity of the relationship between continuous variables and the logit of the outcome was tested using the Box–Tidwell procedure; a significance level of *p* < 0.05 suggested a linear relationship was present. After confirming both the absence of multicollinearity and the linearity assumptions, independent risk factors were selected via stepwise multivariate logistic regression to construct the final nomogram (18). The stepwise backward elimination was indeed performed based on the Akaike information criterion (AIC).

### 2.6 Model evaluation

**Figure 1:**
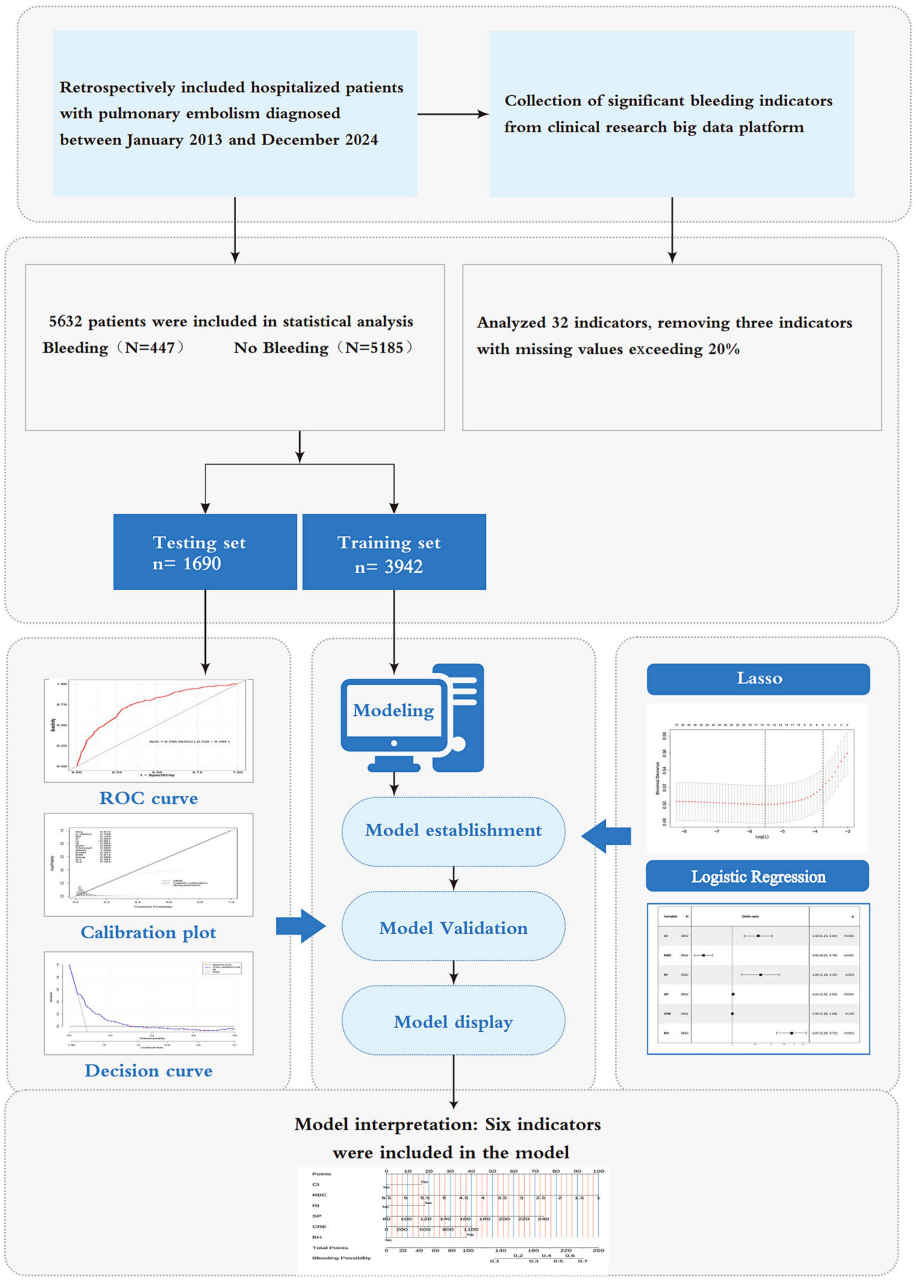
Flowchart of study cohort and prediction model development.

Model performance was comprehensively assessed across three domains: discrimination, calibration, and clinical utility. Discriminatory ability was quantified by the area under the receiver operating characteristic curve (AUC-ROC). Calibration was evaluated through calibration plots. Clinical net benefit across threshold probabilities was analyzed using decision curve analysis (DCA), with additional validation through clinical impact curves (CIC). Finally, the model’s predictive superiority was established by comparing its AUC against individual predictor variables. The complete model training and validation workflow is depicted in Figure 1.

### 2.7 Statistical methods

Statistical analysis and data visualization were performed using R4.4.2 software for Windows. Categorical variables are presented as *n* (%) and were compared using the *χ*^2^ test or Fisher’s exact test. Continuous variables are reported as mean ± standard deviation or median (interquartile range) and were compared using either Student’s *t*-test or the Mann–Whitney *U* test. Multiple imputation techniques were implemented using the “mice” package. Baseline description and difference analysis were performed with the “comparegroups” package. LASSO regression was conducted using the “glmnet” package, while multivariable logistic regression was performed using the “glm” function. Discrimination analysis was carried out using the “pROC,” “ggROC,” and “fbroc” packages. Calibration was assessed using the “rms” and “riskregression” packages. Decision curve analysis (DCA) was conducted using the “rmda” package. The nomogram was created using the “rms” package. Comparisons of multiple models for ROC analysis were conducted using the “ROCR” package. All statistical tests were two-sided, with *p* < 0.05 considered statistically significant.

## 3 Results

### 3.1 Study population characteristics

The study population comprised 5,632 patients with PE, divided into bleeding (*n* = 447) and non-bleeding (*n* = 5,185) cohorts. Significant baseline differences emerged between groups (Table 1). Patients experiencing bleeding events were older (median 77 vs. 74 years, *p* < 0.001) and had higher prevalence of cerebral infarction (48.6% vs. 29.8%), renal insufficiency (24.8% vs. 10.6%), and prior bleeding history (37.8% vs. 13.5%) (all *p* < 0.001). Laboratory parameters revealed the bleeding cohort had lower hemoglobin (97 vs. 109 g/L) and platelet counts (142 vs. 161 × 10^9^/L), but elevated white cell counts (12.78 vs. 10.75 × 10^9^/L) and lactate levels (2.30 vs. 1.90 mmol/L). Vital signs showed elevated blood pressures in bleeding group. Medication analysis indicated more frequent antiplatelet use in the bleeding group (44.3% vs. 32.5%, *p* < 0.001). The training (*n* = 3,942) and testing (*n* = 1,690) sets demonstrated balanced characteristics except for bleeding history prevalence (14.7% vs. 17.2%, *p* = 0.018), suggesting generally representative data partitioning (Table 2).

**Table 1:** Baseline characteristics of subjects.

Variables	Total *N* = 5,632	No bleeding *N* = 5,185	Bleeding *N* = 447	*p*
Age (years)	74.00 [65.75; 82.00]	74.00 [65.00; 81.00]	77.00 [68.00; 83.00]	<0.001
PHT, *n* (%)				0.153
No	5,160 (91.62%)	4,759 (91.78%)	401 (89.71%)	
Yes	472 (8.38%)	426 (8.22%)	46 (10.29%)	
PLT (10^9^/L)	159[121; 200]	161[122; 202]	142 [108; 179]	<0.001
PI, *n* (%)				1.000
No	5,630 (99.96%)	5,183 (99.96%)	447 (100.00%)	
Yes	2 (0.04%)	2 (0.04%)	0 (0.00%)	
CI, *n* (%)				<0.001
No	3,868 (68.68%)	3,638 (70.16%)	230 (51.45%)	
Yes	1,764 (31.32%)	1,547 (29.84%)	217 (48.55%)	
WBC (10^9^/L)	10.88 [7.98; 14.80]	10.75 [7.92; 14.54]	12.78 [8.88; 16.45]	<0.001
HGB (g/L)	108.0 [91.0; 122.0]	109.00 [92.0; 123.0]	97.00 [75.0; 114.5]	<0.001
RBC (10^12^/L)	3.58 [3.05; 4.02]	3.60 [3.10; 4.04]	3.23 [2.52; 3.72]	<0.001
Lac (mmol/L)	2.00 [1.40; 2.80]	1.90 [1.40; 2.80]	2.30 [1.70; 3.45]	<0.001
Syncope, *n* (%)				0.055
No	5,311 (94.30%)	4,899 (94.48%)	412 (92.17%)	
Yes	321 (5.70%)	286 (5.52%)	35 (7.83%)	
RI, *n* (%)				<0.001
No	4,973 (88.30%)	4,637 (89.43%)	336 (75.17%)	
Yes	659 (11.70%)	548 (10.57%)	111 (24.83%)	
DP (mmHg)	99 [93; 110]	99.00 [93; 109]	107 [98; 117]	<0.001
SP (mmHg)	169.0 [155.0; 186.0]	168 [154.0; 184.0]	181 [166.5; 196.0]	<0.001
Tumor, *n* (%)				0.238
No	4,379 (77.75%)	4,021 (77.55%)	358 (80.09%)	
Yes	1,253 (22.25%)	1,164 (22.45%)	89 (19.91%)	
Shock, *n* (%)				<0.001
No	5,396 (95.81%)	4,984 (96.12%)	412 (92.17%)	
Yes	236 (4.19%)	201 (3.88%)	35 (7.83%)	
MI, *n* (%)				0.699
No	5,490 (97.48%)	5,056 (97.51%)	434 (97.09%)	
Yes	142 (2.52%)	129 (2.49%)	13 (2.91%)	
APTT(s)	45.20 [40.10; 52.50]	45.00 [40.00; 52.20]	47.40 [42.25; 56.50]	<0.001
INR	1.34 [1.14; 2.26]	1.33 [1.14; 2.24]	1.44 [1.21; 2.50]	<0.001
PT(s)	15.90 [14.40; 21.10]	15.80 [14.30; 20.80]	17.00 [15.00; 23.30]	<0.001
CRE (μmoI/L)	80.00 [65.00; 104.00]	79.00 [65.00; 102.00]	93.00 [71.50; 134.00]	<0.001
BH, *n* (%)				<0.001
No	4,762 (84.55%)	4,484 (86.48%)	278 (62.19%)	
Yes	870 (15.45%)	701 (13.52%)	169 (37.81%)	
DM, *n* (%)				0.559
No	4,760 (84.52%)	4,387 (84.61%)	373 (83.45%)	
Yes	872 (15.48%)	798 (15.39%)	74 (16.55%)	
HT, *n* (%)				<0.001
No	2,630 (46.70%)	2,469 (47.62%)	161 (36.02%)	
Yes	3,002 (53.30%)	2,716 (52.38%)	286 (63.98%)	
AC, *n* (%)				1.000
No	362 (6.43%)	333 (6.42%)	29 (6.49%)	
Yes	5,270 (93.57%)	4,852 (93.58%)	418 (93.51%)	
TBT, *n* (%)				0.654
No	5,516 (97.94%)	5,080 (97.97%)	436 (97.54%)	
Yes	116 (2.06%)	105 (2.03%)	11 (2.46%)	
Smoking, *n* (%)				0.941
No	3,210 (57.00%)	2,958 (57.05%)	252 (56.38%)	
Yes	2,622 (43.00%)	2,127 (42.95%)	195 (43.62%)	
Drinking, *n* (%)				0.061
No	3,234 (57.42%)	2,993 (57.72%)	241 (53.91%)	
Yes	2,398 (42.58%)	2,192 (42.28%)	206 (46.09%)	
AL, *n* (%)				0.033
No	5,236 (92.97%)	4,832 (93.19%)	404 (90.38%)	
Yes	396 (7.03%)	353 (6.81%)	43 (9.62%)	
AT, *n* (%)				<0.001
No	3,751 (66.60%)	3,502 (67.54%)	249 (55.70%)	
Yes	1,881 (33.40%)	1,683 (32.46%)	198 (44.30%)	

PHT, pulmonary hypertension; PLT, platelet; PI, pulmonary infarction; CI, cerebral infarction; WBC, white blood cell count; HGB, hemoglobin; RBC, red blood cell count; Lac, lactic acid; RI, renal insufficiency; DP, diastolic pressure; SP, systolic pressure; MI, myocardial infarction; APTT, activated partial thromboplastin time; INR, international normalized ratio; PT, prothrombin time; CRE, creatinine; BH, bleeding history; DM, diabetes mellitus; HT, hypertension; AC, anticoagulants; TBT, thrombolytic therapy; AL, arterial thrombosis; AT, antiplatelet therapy.

**Table 2:** The baseline characteristics of the training and testing set.

Variables	Total *N* = 5,632	Testing *N* = 1,690	Training *N* = 3,942	*p*
Age (years)	74.0 [65.8; 82.0]	74.0 [65.0; 81.0]	74.0 [66.0; 82.0]	0.359
PHT, *n* (%)				0.338
No	5,160 (91.6%)	1,558 (92.2%)	3,602 (91.4%)	
Yes	472 (8.4%)	132 (7.8%)	340 (8.6%)	
PLT (10^9^/L)	159 [121; 200]	158.0 [119; 200]	160 [122; 200]	0.453
PI, *n* (%)				1.000
No	5,630 (100.0%)	1,690 (100.0%)	3,940 (99.9%)	
Yes	2 (<0.1%)	0 (0.0%)	2 (0.1%)	
CI, *n* (%)				0.264
No	3,868 (68.7%)	1,179 (69.8%)	2,689 (68.2%)	
Yes	1,764 (31.3%)	511 (30.2%)	1,253 (31.8%)	
WBC (10^9^/L)	10.9 [8.0; 14.8]	10.9 [8.0; 14.7]	10.9 [8.0; 14.8]	0.394
HGB (g/L)	108.0 [91.0; 122.0]	109.0 [92.0; 123.0]	108.0 [90.0; 122.0]	0.235
RBC (10^12^/L)	3.6 [3.0; 4.0]	3.6 [3.1; 4.0]	3.6 [3.0; 4.0]	0.457
Lac (mmol/L)	2.0 [1.4; 2.8]	1.9 [1.4; 2.7]	2.0 [1.4; 2.8]	0.087
Syncope, *n* (%)				0.632
No	5,311 (94.3%)	1,598 (94.6%)	3,713 (94.2%)	
Yes	321 (5.7%)	92 (5.4%)	229 (5.8%)	
RI, *n* (%)				0.231
No	4,973 (88.3%)	1,506 (89.1%)	3,467 (88.0%)	
Yes	659 (11.7%)	184 (10.9%)	475 (12.0%)	
DP (mmHg)	99 [93; 110]	99 [93; 109]	100 [93; 110]	0.242
SP (mmHg)	169 [155; 186]	168 [155; 185]	169 [154; 186]	0.727
Tumor, *n* (%)				0.862
No	4,379 (77.8%)	1,317 (77.9%)	3,062 (77.7%)	
Yes	1,253 (22.2%)	373 (22.1%)	880 (22.3%)	
Shock, *n* (%)				0.921
No	5,396 (95.8%)	1,618 (95.7%)	3,778 (95.8%)	
Yes	236 (4.2%)	72 (4.3%)	164 (4.2%)	
MI, *n* (%)				0.564
No	5,490 (97.5%)	1,651 (97.7%)	3,839 (97.4%)	
Yes	142 (2.5%)	39 (2.3%)	103 (2.6%)	
APTT(s)	45.2 [40.1; 52.5]	45.3 [40.2; 52.5]	45.2 [40.1; 52.6]	0.950
INR	1.3 [1.1; 2.3]	1.3 [1.1; 2.3]	1.3 [1.1; 2.3]	0.740
PT(s)	15.9 [14.4; 21.1]	15.9 [14.4; 21.4]	15.9 [14.4; 21.0]	0.888
CRE (μmoI/L)	80.0 [65.0; 104.0]	79.0 [65.0; 103.0]	80.0 [65.0; 104.0]	0.320
BH, *n* (%)				0.018
No	4,762 (84.6%)	1,399 (82.8%)	3,363 (85.3%)	
Yes	870 (15.4%)	291 (17.2%)	579 (14.7%)	
DM, *n* (%)				0.738
No	4,760 (84.5%)	1,433 (84.8%)	3,327 (84.4%)	
Yes	872 (15.5%)	257 (15.2%)	615 (15.6%)	
HT, *n* (%)				0.893
No	2,630 (46.7%)	792 (46.9%)	1,838 (46.6%)	
Yes	3,002 (53.3%)	898 (53.1%)	2,104 (53.4%)	
AC, *n* (%)				0.398
No	362 (6.4%)	101 (6.0%)	261 (6.6%)	
Yes	5,270 (93.6%)	1,589 (94.0%)	3,681 (93.4%)	
TBT, *n* (%)				0.244
No	5,516 (97.9%)	1,649 (97.6%)	3,867 (98.1%)	
Yes	116 (2.1%)	41 (2.4%)	75 (1.9%)	
Smoking, *n* (%)				0.253
No	3,210 (57.0%)	935 (55.3%)	2,275 (57.7%)	
Yes	2,422 (43.0%)	755 (44.7%)	1,667 (42.3%)	
Drinking, *n* (%)				0.857
No	3,234 (57.4%)	961 (56.9%)	2,273 (57.7%)	
Yes	2,398 (42.6%)	729 (43.1%)	1,669 (42.3%)	
AL, *n* (%)				0.344
No	5,236 (93.0%)	1,580 (93.5%)	3,656 (92.7%)	
Yes	396 (7.0%)	110 (6.5%)	286 (7.3%)	
AT, *n* (%)				0.582
No	3,751 (66.6%)	1,135 (67.2%)	2,616 (66.4%)	
Yes	1,881 (33.4%)	555 (32.8%)	1,326 (33.6%)	

PHT, pulmonary hypertension; PLT, platelet; PI, pulmonary infarction; CI, cerebral infarction; WBC, white blood cell count; HGB, hemoglobin; RBC, red blood cell count; Lac, lactic acid; RI, renal insufficiency; DP, diastolic pressure; SP, systolic pressure; MI, myocardial infarction; APTT, activated partial thromboplastin time; INR, international normalized ratio; PT, prothrombin time; CRE, creatinine; BH, bleeding history; DM, diabetes mellitus; HT, hypertension; AC, anticoagulants; TBT, thrombolytic therapy; AL, arterial thrombosis; AT, antiplatelet therapy.

### 3.2 Selected predictors and construction model

**Figure 2:**
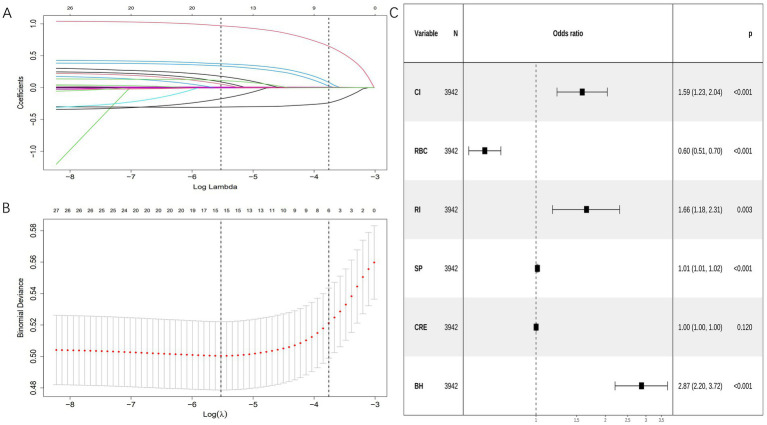
Variable selection was performed using LASSO and logistic regression. **(A)** Coefficient profile plots were generated against the log(lambda) sequence to visualize the variable selection process and identify nonzero coefficient variables based on the optimal lambda value. **(B)** Dotted vertical lines represent optimal values determined using the 1 standard error of the minimum criteria (lambda.1se). **(C)** Forest plot displaying final predictors in the bleeding risk model with adjusted odds ratios from multivariable logistic regression. CI, cerebral infarction; RBC, red blood cell count; RI, renal insufficiency; SP, systolic pressure; CRE, creatinine; BH, bleeding history.

Variable selection was performed using LASSO regression with tenfold cross-validation, which identified six clinically significant predictors: cerebral infarction, red blood cell count, renal insufficiency, systolic pressure, creatinine, and bleeding history. The regularization path showing coefficient shrinkage is presented in Figure 2A, with optimal lambda selection demonstrated in Figure 2B. The results showed that the included variables had no collinearity in predicting respiratory failure (VIFs <10), and there was a linear relationship with logitp (*p* > 0.05), suggesting that they could be used to construct a logistic regression model. All selected variables were subsequently incorporated into a multivariable logistic regression model using backward elimination (minimum AIC = 1,972). The final model retained six significant predictors (Table 3 and Figure 2C).

**Table 3:** Final model coefficients.

Characteristics	B	SE	OR	CI	*p*
(Intercept)	−3.649	0.5698	0.026	0.008–0.079	<0.001
CI	0.461	0.12816	1.586	1.233–2.038	<0.001
RBC	−0.512	0.08206	0.599	0.509–0.703	<0.001
RI	0.504	0.17126	1.656	1.178–2.307	0.003
SP	0.013	0.00272	1.013	1.007–1.018	<0.001
CRE	0.001	0.00066	1.001	0.999–1.002	0.12
BH	1.054	0.13355	2.869	2.203–3.720	<0.001

CI, cerebral infarction; RBC, red blood cell count; RI, renal insufficiency; SP, systolic pressure; CRE, creatinine; BH, bleeding history.

### 3.3 Model visualization

**Figure 3:**
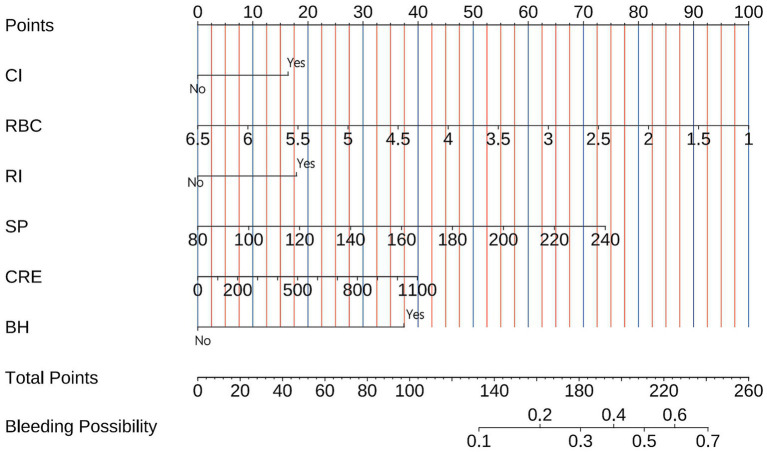
Nomogram for bleeding risk prediction in pulmonary embolism patients. The tool converts six clinical parameters into points: cerebral infarction (Cl), red blood cell count (RBC), renal insufficiency (RI), systolic blood pressure (SP), creatinine (CRE), and bleeding history (BH). Summed points (total points axis) correspond to predicted bleeding probability (bottom axis). Example: A patient with prior bleeding (bleeding history = yes, 37.5 points), cerebral infarction (CI = yes, 16 points), renal insufficiency (RI = yes, 18 points), RBC 2.5 × 10^12^/L (73 points), systolic pressure 160 mmHg (37 points), and creatinine 500 μmol/L (18 points) would have 199.5 points, corresponding to 44% bleeding risk.

The final bleeding risk prediction model was operationalized through a clinically deployable nomogram (Figure 3). This visual tool integrates six significant predictors identified during model development. Each predictor is assigned points along scaled axes according to its regression weight. Clinicians sum the points corresponding to a patient’s clinical profile, with the total points axis (0–260 points) providing immediate conversion to predicted bleeding probability (0.1–0.7). For example: A patient with prior bleeding (bleeding history = yes, 37.5 points), cerebral infarction (CI = yes, 16 points), renal insufficiency (RI = yes, 18 points), RBC 2.5 × 10^12^/L (73 points), systolic pressure 160 mmHg (37 points), and creatinine 500 μmol/L (18 points) would have 199.5 points, corresponding to 44% bleeding risk.

### 3.4 Model validation

**Figure 4:**
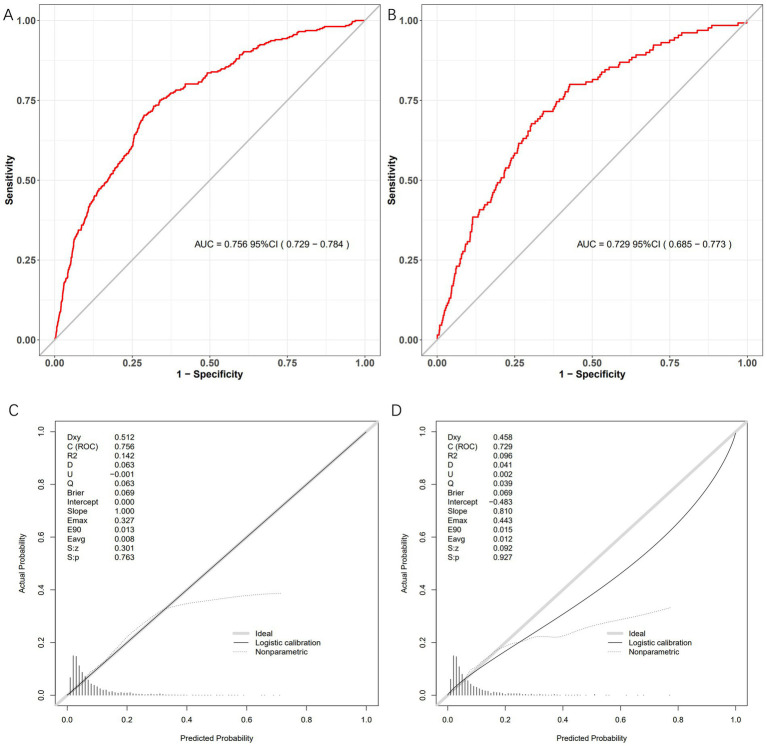
Model performance metrics in development and validation cohorts. Four-panel evaluation of the bleeding risk prediction model. **(A)** Development ROC: AUC = 0.756 (95% CI: 0.729–0.784). **(B)** Validation ROC: AUC = 0.729 (95% CI: 0.685–0.773). **(C)** Development calibration: Ideal fit (slope = 1.000). **(D)** Validation calibration: Good agreement (slope = 0.810).

**Figure 5:**
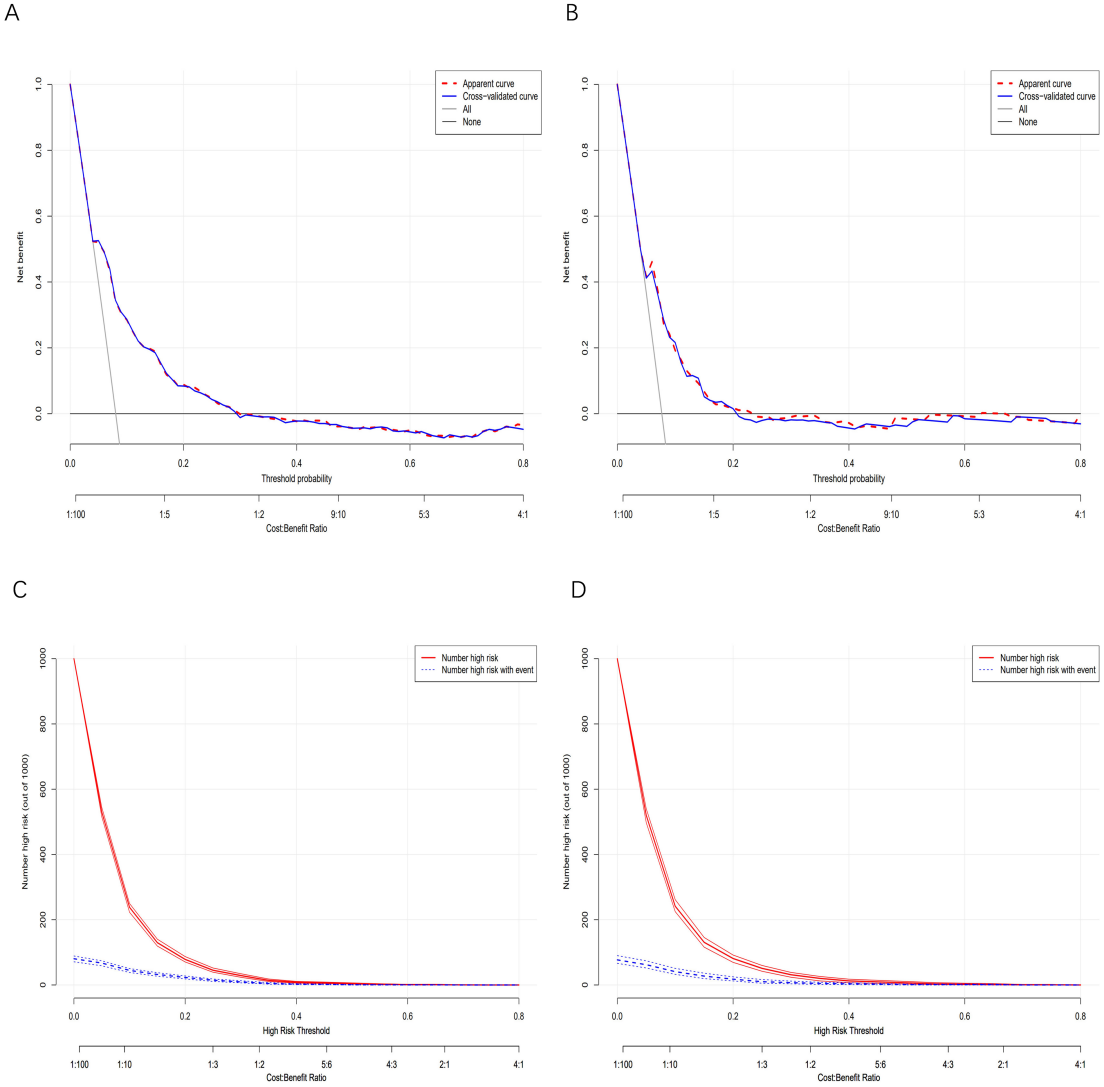
Clinical utility and impact analysis. **(A)** Development DCA: Net benefit of model-guided decisions versus “treat-all” and “treat-none” strategies across threshold probabilities. **(B)** Validation DCA: Replication of net benefit superiority in independent cohort. **(C)** Development CIC: Proportion classified high-risk versus actual bleeding events captured. **(D)** Validation CIC: Replication of clinical impact in independent cohort. In the DCAs, the *y*-axis represents the net benefit. The horizontal lines labeled “None” represent the assumption that no participant experienced bleeding. The lines labeled “All” represent the assumption that all participants had bleeding. The lines labeled “nomogram model” represent the predictive model developed in this study. In CICs, the red curve represents the number of individuals classified as positive (high risk) by the model at each threshold probability, indicating the number of high-risk individuals. The blue curve represents the number of true positives (individuals with the outcome) at each threshold probability.

The bleeding risk model demonstrated robust performance in both training and validation cohorts. In Figure 4A, the AUC of the training cohort was 0.756 (95% CI: 0.729–0.784), while in Figure 4B, the AUC of the validation cohort was 0.729 (95% CI: 0.685–0.773). Both significantly exceeded the null hypothesis value of 0.5 (*p* < 0.001), confirming clinically useful discriminatory power. Calibration curves (Figures 4C,D) illustrate the excellent concordance between the predicted probability of bleeding and the actual observations in the training and validation cohort. Brier scores were low and consistent (training: 0.069; validation: 0.069), indicating stable predictive accuracy. Decision curve analysis demonstrated robust clinical utility across cohorts. In the training cohort, the model provided superior net benefit versus default strategies across threshold probabilities 5–35% (Figure 5A), with optimal clinical utility at 5% risk where net benefit reached 0.52. Validation cohort maintained significant net benefit (Figure 5B), particularly at critical thresholds 5–26% (maximum NB = 0.42 at 10% risk). Clinical impact curves demonstrated consistent risk stratification utility across cohorts. In the training cohort (Figure 5C), at the 30% probability threshold: 31.2% (1,230/3,942) of patients were classified as high-risk, capturing 78.5% (351/447) of bleeding events (sensitivity) with a positive predictive value (PPV) of 28.5% (351/1,230), translating to 1 true positive identified per 3.5 high-risk patients treated. Validation cohort (Figure 5D) analysis confirmed robustness: at 30% threshold, 28.6% (484/1,690) were high-risk, detecting 76.3% (65/85) of bleeding events (PPV = 13.4%), requiring treatment of 7.4 patients per true bleed prevented.

### 3.5 Model compare with single indicator

**Figure 6:**
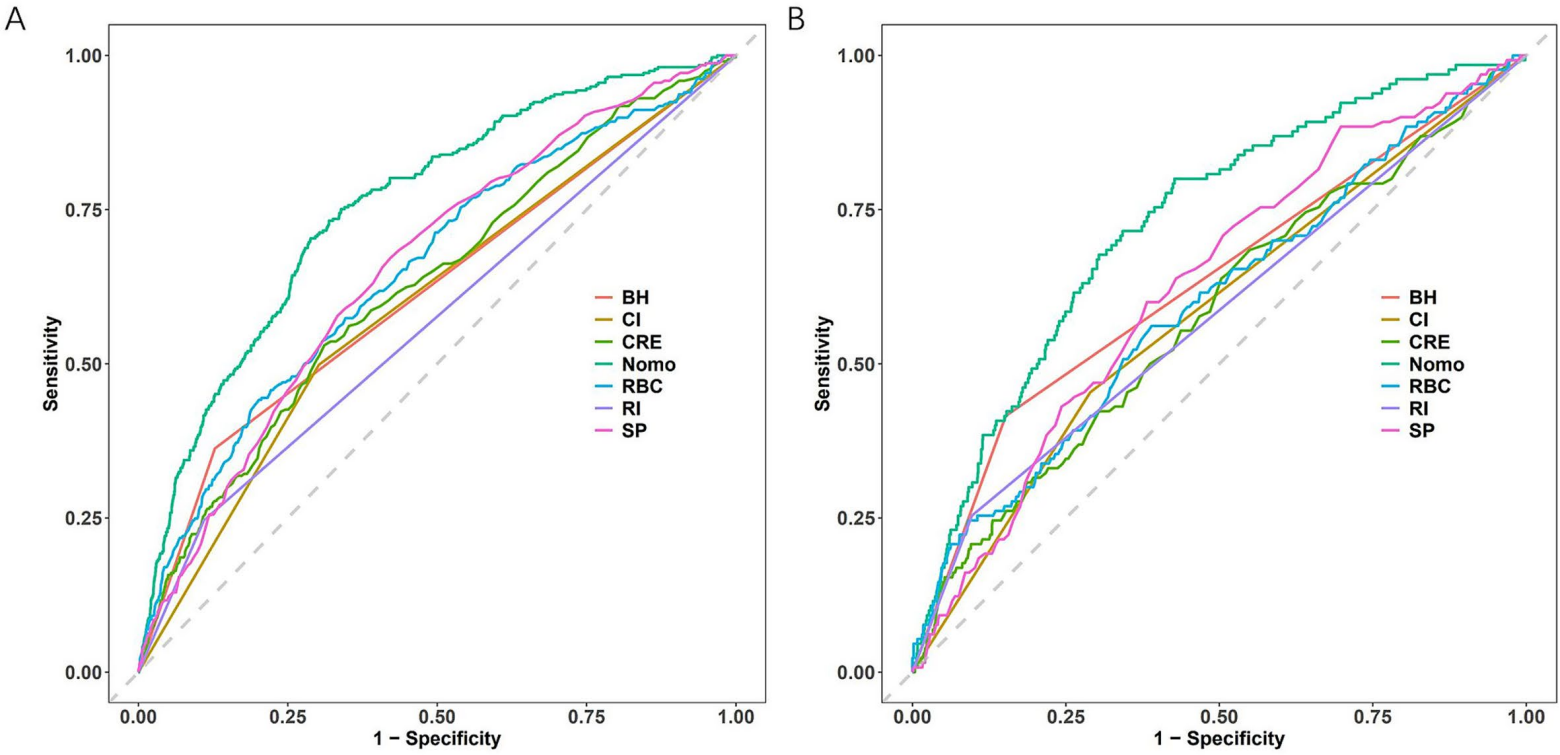
ROC curve comparisons. **(A)** Training cohort. **(B)** Testing cohort. Nomogram has the maximum AUC. CI, cerebral infarction; RBC, red blood cell count; RI, renal insufficiency; SP, systolic pressure; CRE, creatinine; BH, bleeding history; Nomo, nomogram.

The nomogram demonstrated superior discriminatory capacity compared to individual predictors in both training and validation cohorts (Figure 6). In the training cohorts (Figure 6A), the nomogram model achieved significantly higher AUC (0.756, 0.729–0.784) than any single predictor (*p* < 0.01 for all comparisons). Validation cohort results (Figure 6B) confirmed this superiority, model AUC remained robust at 0.729 (95% CI: 0.685–0.773).

## 4 Discussion

This study developed and rigorously validated a novel bleeding risk prediction model for PE patients using LASSO regression. Clinicians can utilize this validated model incorporating six key predictors (bleeding history, renal function, RBC count, blood pressure, stroke history, and creatinine levels), which demonstrated reliable risk stratification (development/validation AUCs: 0.756/0.729) and accurate probability estimation. The resultant nomogram provides clinicians with an individualized risk quantification tool that translates complex model outputs into actionable bedside decisions.

In patients with PE undergoing anticoagulation therapy, bleeding is a major complication. Studies show that a history of bleeding is a significant factor influencing the risk of bleeding. In one study, a history of bleeding was identified as a significant risk factor for major bleeding in PE patients receiving thrombolysis (19). Our study reaffirmed the importance of history of bleeding as a critical indicator for assessing bleeding risk. Additionally, renal insufficiency is a key factor affecting the risk of bleeding. Research has found that renal insufficiency, particularly acute kidney injury (AKI) and severe renal insufficiency, is significantly associated with early mortality in acute PE patients (20). Another study found that patients with renal insufficiency have a higher incidence of bleeding events during hospitalization, especially when using conventional doses of low molecular weight heparin (LMWH) (21). Additionally, serum creatinine and estimated glomerular filtration rate (eGFR) are also important indicators of long-term outcomes. Studies indicate that decreased renal function is associated with an increased risk of all-cause mortality 90 days and 1 year after acute PE, underscoring the importance of monitoring renal function in managing patients with PE (22). Our predictive model aligned with previous studies’ findings. RBC is also a factor that influences the risk of bleeding. Studies have shown that red blood cell distribution width (RDW) is significantly associated with the mortality rate in patients with PE, and an elevated RDW may indicate a poor prognosis (23). Our findings indicated that RBC is a significant factor that elevates the risk of bleeding. Additionally, inflammatory markers such as interleukin-8 (IL-8) have been linked to early major bleeding in patients with acute PE, suggesting that these biomarkers may play a role in future risk assessments (24). Research has shown that hypertension and systolic blood pressure play a significant role in the impact on patients with PE. Studies indicate that in hypertensive patients treated with fibrinolytic therapy, systolic blood pressure levels are significantly associated with the occurrence of cerebral hemorrhage (25). Our study uncovered a substantial increase in bleeding risk associated with hypertension. This elevated risk may be attributed to structural changes in blood vessels caused by hypertension, rendering them more susceptible to rupture and ultimately increasing the likelihood of bleeding (26). Artery occlusion cerebral infarction was associated with an elevated risk of hemorrhage transformation (27), consistent with our results and potentially linked to increased antithrombotic medication usage.

When assessing the bleeding risk in patients with PE, using existing scoring systems can be helpful. However, studies have shown that current scoring systems are not sufficiently accurate in predicting early major bleeding in patients with acute PE, necessitating the development of a specific risk scoring system for acute PE (4). Our study delivers three pivotal contributions to personalized PE management: First, we establish the first bleeding risk prediction model specifically derived for PE populations, overcoming critical limitations of generic thrombotic risk tools. By employing LASSO regression to integrate six evidence-based predictors (prior hemorrhage, renal dysfunction, erythrocyte count, systolic hypertension, cerebral infarction, and creatinine), our model addresses the unmet need for PE-specific risk stratification. Second, the clinically deployable nomogram transforms complex algorithmic outputs into immediate, individualized risk quantitation—enabling dynamic optimization of anticoagulation intensity, comorbidity management (e.g., hypertension control), and hematologic parameter correction at point-of-care. Third, decision curve and clinical impact analyses demonstrate significant net benefit improvement across critical thresholds, substantiating its capacity to reduce major bleeding events in high-risk subgroups.

Several limitations merit acknowledgment. First, the 6-month observation period limits assessment of long-term bleeding risk. Second, the single-center retrospective design fundamentally restricts the ability to perform a direct comparison with conventional risk scores, despite our efforts to mitigate bias. Third, and most importantly, the model’s performance in ethnically diverse populations and different healthcare systems is unknown and represents a critical question for future research.

Our study establishes and validates a new pulmonary embolism-specific bleeding risk prediction model. The resultant nomogram demonstrated robust discrimination and calibration, this tool enables personalized anticoagulation intensity adjustment and targeted comorbidity management. Future implementation in multinational pragmatic trials will validate its capacity to improve patient outcomes while reducing healthcare utilization costs.

## Data Availability

The raw data supporting the conclusions of this article will be made available by the authors, without undue reservation.
